# TLR2 and Nod2 Mediate Resistance or Susceptibility to Fatal Intracellular *Ehrlichia* Infection in Murine Models of Ehrlichiosis

**DOI:** 10.1371/journal.pone.0058514

**Published:** 2013-03-19

**Authors:** Partho Chattoraj, Qin Yang, Ankita Khandai, Omar Al-Hendy, Nahed Ismail

**Affiliations:** Department of Pathology, University of Pittsburgh, Pittsburgh, Pennsylvania, United States of America; Washington State University, United States of America

## Abstract

Our murine models of human monocytic ehrlichiosis (HME) have shown that severe and fatal ehrlichiosis is due to generation of pathogenic T cell responses causing immunopathology and multi-organ failure. However, the early events in the liver, the main site of infection, are not well understood. In this study, we examined the liver transcriptome during the course of lethal and nonlethal infections caused by *Ixodes ovatus Ehrlichia* and *Ehrlichia muris*, respectively. On day 3 post-infection (p.i.), although most host genes were down regulated in the two groups of infected mice compared to naïve counterparts, lethal infection induced significantly higher expression of *caspase* 1, *caspase 4*, nucleotide binding oligomerization domain-containing proteins (*Nod1*), tumor necrosis factor-alpha, interleukin 10, and *CCL7* compared to nonlethal infection. On day 7 p.i., lethal infection induced highly significant upregulation of type-1 interferon, several inflammatory cytokines and chemokines, which was associated with increased expression levels of Toll-like receptor-2 (*TLR2*), *Nod2*, *MyD88*, nuclear factor-kappa B (*NF-kB*), *Caspase 4*, *NLRP1*, *NLRP12*, *Pycard*, and *IL-1β*, suggesting enhanced TLR signals and inflammasomes activation. We next evaluated the participation of TLR2 and Nod2 in the host response during lethal *Ehrlichia* infection. Although lack of TLR2 impaired bacterial elimination and increased tissue necrosis, Nod2 deficiency attenuated pathology and enhanced bacterial clearance, which correlated with increased interferon-γ and interleukin-10 levels and a decreased frequency of pathogenic CD8^+^ T cells in response to lethal infection. Thus, these data indicate that Nod2, but not TLR2, contributes to susceptibility to severe *Ehrlichia*-induced shock. Together, our studies provide, for the first time, insight into the diversity of host factors and novel molecular pathogenic mechanisms that may contribute to severe HME.

## Introduction

Human monocytic ehrlichiosis (HME) is an emerging tick-borne disease caused by *Ehrlichia chaffeensis*, a Gram-negative obligate intracellular bacterium that lacks lipopolysaccharide (LPS) [1]–[4]. *E. chaffeensis* causes pancytopenia and hepatic dysfunction, which progress to a potentially fatal multiorgan system disorder that mimics toxic shock syndrome despite antibiotic treatment [5]–[8]. Ehrlichiae infect several myeloid cells, such as macrophages, monocytes, and dendritic cells, and thus cause systemic infection [2], [3], [9]–[12]. In mice, innocuous or fatal ehrlichial diseases that mimic different spectra of HME occur following infection with *E. muris* and *Ixodes ovatus Ehrlichia* (IOE), respectively [13]–[15]. These two *Ehrlichia* species are not only genetically and antigenically related to *E. chaffeensis* but also cause human infections [14], [15]. Lethal *Ehrlichia* infection is characterized by extensive tissue damage in the absence of overwhelming infection, suggesting an immune-mediated pathology [16]–[20]. Protective immunity against *Ehrlichia* is mediated by interferon (IFN)-γ production by CD4^+^ T helper (Th)1 cells and natural killer T (NKT) cells [20]–[22]. However, these cells undergo apoptosis at late stages of severe infection [17], [23]. Recently, we demonstrated that cytotoxic and cytokine-producing NK and CD8^+^ T cells mediate tissue injury and impair anti-*Ehrlichia* protective immunity during lethal *Ehrlichia* infection [20], [23].

Innate immune cells express many pattern recognition receptors (PRRs) that are activated upon recognition of pathogen-associated molecular patterns [24]–[28]. The most characterized PRRs are the TLRs, which are transmembrane proteins localized either at the cell surface or within endosomal membranes. Upon activation, these receptors initiate signaling pathways dependent on adaptor proteins, such as MyD88, that result in activation of nuclear factor-kappa B (NF-κB) [26]–[30]. Other intracellular PRRs that emerged as sensors for intracellular microbial infection are the nucleotide-binding oligomerization domain (Nod)-like receptor protein (NLR) family, which includes Nod1 and Nod2 [31]–[34]. Nod1 and Nod2 signal via the adaptor molecule Rip2, a protein kinase required for activation of NF-κB and MAPK cascades, resulting in production of many cytokines and chemokines. Nod1 and Nod2 activation are upstream sensory signals for activation of the inflammasomes in the cytosol, which forms only in response to danger signals, including bacterial or viral infection. Activation of the inflammasomes leads to cleavage of caspase 1, which in turn cleaves pro-interleukin (IL)-1β and pro-IL-18, producing biologically active IL-1β and IL-18 [35]–[38]. These cytokines play different roles in inflammation and host defense against pathogens [39]–[42]. Currently, there are four defined inflammasomes. NLRP3 and Nalp1 trigger activation in response to extracellular adenosine-5'-triphosphate and pore-forming toxins [43], [44]. NLRC4 is able to recognize many bacterial proteins found in the bacterial type III secretion apparatus [35]–[38]. Absent in melanoma 2 is able to sense cytosolic double-stranded DNA [45].

The first objective of this study was to better understand the pattern of gene expression underlying immune responses against *Ehrlichia* during tissue damage or recovery following lethal or nonlethal infections, respectively. Our results suggest that genes with specific biologic functions, including inflammasomes, TLR2 and Nod2, and several cytokines and chemokines are differentially regulated during mild and severe ehrlichiosis. The second objective is to examine the contribution of Nod2 or TLR2 to host defense against *Ehrlichia* and pathogenesis of HME. Strikingly, we found that TLR2-dependent host responses contribute to protective immunity against *Ehrlichia*. In contrast, Nod2-dependent host responses negatively regulate anti-*Ehrlichia* protective immunity and promote the development of pathogenic immune responses, thus enhancing susceptibility to *Ehrlichia*-induced toxic shock.

## Materials and Methods

### Ethics Statement

This study was carried out in strict accordance with the recommendations in the Guide for the Care and Use of Laboratory Animals of the National Institutes of Health. The protocol was approved by the Committee on the Ethics of Animal Experiments of the University of Pittsburgh in accordance with the institutional guidelines for animal welfare.

### Mice and *Ehrlichia* infection

Female C57BL/6J, B6.129-Tlr2^tm1Kir^/J, and B6.129S1-Nod2^tm1Flv^/J mice of 8–12 weeks of age were obtained from Jackson Laboratories (Bar Harbor, ME). All animals were housed under specific pathogen-free conditions at the Animal Research Facility in the University of Pittsburgh. Two species of monocytic *Ehrlichia* were used in this study: the highly virulent IOE and the mildly virulent *E. muris*. IOE and *E. muris* stocks were propagated by passage through wild type C57BL/6 mice. Single-cell suspensions from spleens harvested from mice 7 days post-infection (p.i.) were stored in sucrose and potassium phosphate (SPK) buffer (0.5 M K_2_HPO_4_, 0.5 M KH_2_PO_4_, and 0.38 M sucrose) in liquid nitrogen and used as stocks. Mice were infected intraperitoneally (i.p.) with a lethal high dose of IOE (10^4^ organisms/mouse) or a nonlethal high dose of *E. muris* (2 × 10^5^ organism/mouse). Mice were monitored daily for signs of illness and survival.

### Reverse transcription and real-time polymerase chain reaction (RT-PCR) arrays

Quantitative RT-PCR was carried out for groups of genes that are involved with different functions, such as immune regulation, innate and adaptive immune responses, and host cell survival. RNA was isolated from liver tissues using the Ambion RNA isolation kit (Life Technologies, Grand Island, NY), and cDNA was synthesized using the SA Biosciences RT^2^ First Strand Kit (QIAGEN, Valencia, CA) following the manufacturer’s recommendations. The expression levels of ∼ 200 genes were determined using SA Bioscience Pathway Finder RT^2^ Profiler^Tm^ PCR arrays for apoptosis, inflammasomes, cytokines, and innate and adaptive immune responses following the manufacturer’s recommendations. Data were collected using an Applied Biosystems 7900 HT Real-Time PCR System. The array plate contained 5 house-keeping genes, including GAPDH and β-actin, and one set for genomic DNA contamination as reference genes and a control. Comparative threshold cycle values were analyzed using SA Biosciences software, and fold regulation values were plotted. Fold regulation values were calculated by dividing the expression fold changes of the candidate genes by the expression fold changes of the reference genes using the comparative threshold cycle method. Upregulation or down regulation of host genes was determined based on comparison with naïve mice. Using cut-off criteria, a 5-fold upregulation or downregulation was considered to be significant and of biologic importance.

### Flow cytometry

Splenocytes were harvested, counted, and resuspended in staining buffer at a concentration of 10^6^ cells/tube. FcRs were blocked with a mAb (clone 2.4G2) against mouse CD16 and CD32 for 15 min. The following fluorescein isothiocyanate (FITC)-, phycoerythrin (PE)-, PerCP-Cy5.5-, Alexa Fluor-, and allophycocyanin-conjugated monoclonal antibodies (mAb) were purchased from BD Biosciences: anti-CD3 (clone 145- 2C11), anti-CD4 (clone RM4-4), anti-CD8α (clone 53-6.7), and anti-NK1.1 (clone PK136). Appropriate isotype control mAb, including FITC-, PE-, or allophycocyanin-conjugated hamster IgG1 (A19-3), rat IgG1 (R3-34), rat IgG2a (R35-95), mouse IgG1 (X40), and rat IgG2b (A95-1) were purchased from BioLegend (San Diego, CA). Lymphocyte and granulocyte populations were gated based on forward and side scatter parameters; 20,000–50,000 events were collected using BD-LSR or BD FACSCalibur (BD Immunocytometry Systems, San Jose, CA) flow cytometers. Data were analyzed using FlowJo software (TreeStar, Ashland, OR).

### In vitro splenocyte stimulation and cytokine enzyme-linked immunosorbent assay (ELISA)

Spleens were harvested and single-cell suspensions were prepared as described before [19]–[21]. A total of 2–5×10^6^ cells were seeded into a 12-well tissue culture plate in RPMI, supplemented with 10% heat-inactivated fetal bovine serum, 1% HEPES buffer, and 100 µg/ml penicillin and streptomycin. Splenocytes were cultured with and without IOE antigens. After 48 hours, the culture supernatants were collected and an IFN-γ concentration was determined using the mouse Quantikine ELISA kit (R&D Systems, Minneapolis, MN) according to the manufacturer’s recommendations. The minimum detection limit for IFN-γ is 2 pg/ml.

### Bacterial burden determination using real-time PCR

Total DNA was isolated from liver and spleen tissues using the DNeasy Blood and Tissue kit (QIAGEN). Bacterial burden was determined using a Step One Plus Real-Time PCR machine (Life Technologies, Grand Island, NY) targeting the EM/IOE *dsb* gene as previously described (16). The primers and probes used are as follows: EM/IOE *dsb*-F: 5′-CAG GAT GGT AAA GTA CGT GTG A-3′; EM/IOE *dsb*-R: 5′- TAG CTA AYG CTG CCT GGA CA-3′; EM/IOE probe: (6FAM)-AGG GAT TTC CCT ATA CTC GGT GAG GC-(MGB-BHQ). The eukaryotic housekeeping gene *gapdh* was amplified using the following primers/probes: GAPDH-F: 5′-CAA CTA CAT GGT CTA CAT GTT C-3′; GAPDH-R: 5′-TCG CTC CTG GAA GAT G-3'; GAPDH probe: (6FAM)-CGG CAC AGT CAA GGC CGA GAA TGG GAA GC-(MGB-BHQ). The comparative cycle threshold method was used to determine the bacterial burden as described previously [19]. The results were normalized to the levels of expression of the *gapdh* in the same sample and expressed as copy number per 10^4^ copies of *gapdh* [16], [38]. PCR analyses were considered negative for ehrlichial DNA if the critical threshold values exceeded 40 cycles.

### Histopathology and terminal deoxynucleotidyl transferase dUTP nick end labeling (TUNEL) assay

Tissue sections were fixed in a 10% solution of neutral buffered formalin, dehydrated in graded alcohols, embedded in paraffin wax, and stained with hematoxylin and eosin (H&E). Semi-quantitative analysis of the liver lesions was carried out using three parameters: the number of necrotic cells, the number of apoptotic cells, and the number of inflammatory foci in each high power field (HPF). TUNEL staining was performed on unstained tissue sections, showing apoptotic cell death without focal necrosis, as described previously.

### Statistical analyses

The two-tailed t test was used for comparisons of mean values for two experimental groups, and one-way analysis of variance was used for comparisons of multiple experimental groups. Data were represented by means ± standard deviations (SDs) or standard errors of the mean. P values P values ≤ 0.001 were considered highly significant (***); P value ≤ 0.01 were considered moderately significant (**), and p values ≤ 0.05 were considered significant (*).

## Results

### Identification of transcripts altered by lethal *Ehrlichia* infection

Previous murine studies indicated that protection against *Ehrlichia* is mediated by IFN-γ and CD4^+^ Th1 cells whereas *Ehrlichia*-induced shock can be attributed to CD4^+^ Th1 hyporesponsiveness and the induction of pathogenic NK and CD8^+^ T cells mediating host cell apoptosis and necrosis [17]–[23]. In this study, we examined the expression of several genes that are involved in host cell survival and innate and adaptive immune responses in the livers of murine models of mild and fatal ehrlichiosis caused by *E. muris* and IOE, respectively. We chose to study the liver for two reasons: 1) the liver is the primary site for *Ehrlichia* infection and pathology in humans and mice [5], [7], [12], [13], and 2) previous studies indicated that the spatial and temporal changes in immune responses in the liver are strong predictors of disease progression in a mouse model of fatal HME [23]. We analyzed gene transcripts relevant to specific pathways, including: apoptosis, inflammasomes, and TLR signaling, and innate and adaptive immune responses. Overall, lethal or nonlethal infections induced significant (p ≤ 0.05) downregulation of several genes on day 3 p.i. On the other hand, the majority of gene transcripts were upregulated on day 7 p.i. with both bacterial species but with more dramatic changes in response to lethal than nonlethal infection (Table 1 and 2).

**Table 1:** Differential gene expression of chemokine and cytokines.

		Post infection day 3		Post infection day 7	
#	Gene Symbol	Non-lethal*/E. muris*	Lethal/*IOE*	Non-lethal/*E. muris*	Lethal/ *IOE*
1	Ccl2	1.1	–1.3	38.7	978.9
2	Ccl3	4.6	26.2	5.7	147.7
3	Ccl4	1.2	13.2	16.6	234.4
4	Ccl5	1.5	1.0	4.9	78.9
5	Ccl6	1.4	15.3	2.5	1.9
6	Ccl7	–1.1	641.5	82.4	726.6
7	Ccl8	–1.1	130.3	15.3	281.3
8	Ccl9	1.7	7.9	3.8	8.2
9	Ccl11	1.2	2.0	2.7	28.3
10	Ccl12	2.6	520.1	48.4	452.1
11	Ccl19	–1.5	6.1	13.1	19.5
12	Ccl24	1.4	16.3	5.5	–4.2
13	CCR2	–1.1	8.9	7.5	3.7
14	Ccr3	67.0	361.4	3.0	2.4
15	Ccr7	–2.2	2.5	2.2	16.2
16	Cxcl1	1.5	1.4	23.6	80.4
17	Cxcl5	1.5	19.9	8.1	14.4
18	Cxcl9	–1.9	221.9	54.9	132.1
19	Cxcl10	–1.2	84.0	27.1	178.0
20	Cxcl11	–27.6	2.8	12.1	130.9
21	Cxcl13	1.8	12.9	2.8	9.3
22	Il1alpha	1.6	15.2	4.5	3.7
23	Il6	–1.2	1.1	5.1	24.9
24	Tnf	1.6	59.3	19.6	52.4
25	Tnfsf11	1.9	2.6	18.6	2.4
26	Tnfrsf10b	3.0	1.8	2.0	15.0
27	Tnfsf10	–1.3	9.3	4.3	8.2
28	Traf1	4.0	13.4	6.9	42.9
29	Traf2	–1.3	–1.1	125.5	2.4
30	Ifnb1	1.1	2.2	2.8	275.7
31	Ifng	4.8	7.1	111.9	154.8
32	Il12a	1.9	2.6	3.3	11.8
33	Il12b	1.5	2.1	19.9	32.7
34	Il10	–1.3	35.2	5.3	43.6
35	Il1ra	2.1	9.4	9.8	181.1

# 1–21 Chemokine and their receptor

# 22–29 Pro-inflammatory cytokines and signaling genes

# 30–33 Type-1 interferons and Th1 cytokines

# 34–35 Anti-inflammatory cytokines

**Table 2:** Differential gene expression of TLR, Inflammasomes, and Apoptosis Receptors.

		Post infection day 3		Post infection day 7	
#	Gene Symbol	Non-lethal*/E. muris*	Lethal/*IOE*	Non-lethal/*E. muris*	Lethal/ *IOE*
1	Cd14	1.4	1.0	3.5	77.5
2	Cd80	–2.1	1.0	5.8	8.8
3	Cd40	–1.4	21.7	17.4	58.3
4	Cd40lg	–1.6	4.5	3.9	14.2
5	Tlr1	–3.4	1.9	3.7	7.4
6	Tlr2	–2.6	–5.4	8.0	62.9
7	Tlr3	–1.9	–1.3	1.7	9.7
8	Tlr9	–2.4	1.2	1.5	16.3
9	Jun	1.2	1.4	3.4	20.9
10	Myd88	–1.6	–1.1	2.2	8.8
11	Nf-kb2	–1.7	–2.5	4.4	7.7
12	Nod1	–1.6	80.8	2.0	1.8
13	Nod2	1.8	2.3	43.2	23.0
14	Ripk2	–1.1	4.4	2.8	6.7
15	Apaf1	1.3	1.5	3.0	10.3
16	Birc2	6.2	14.1	2.0	6.0
17	Ciita	2.1	2.6	122.6	16.6
18	Il1b	1.7	–1.3	2.0	7.8
19	IL-18	1.3	7.3	–1.7	2.8
20	Casp1	–1.5	12.5	14.5	15.7
21	Casp12	–1.9	2.0	6.2	11.3
22	Casp4	2.8	32.8	7.1	26.9
23	Nlrc5	1.3	–1.7	9.0	9.1
24	Nlrp12	–1.4	–1.2	–1.2	18.3
25	Nlrp1	–2.2	–3.6	1.1	6.3
26	Nlrp3	–1.3	–2.0	–3.0	5.0
27	Bcl2	9.9	10.7	6.1	2.8
28	Bcl2l2	2.8	1.1	1.8	40.7
29	Bak1	2.7	10.2	1.4	1.1
30	Bax	3.1	7.3	1.6	2.3
31	Apaf1	1.3	1.5	3.0	10.3
32	AIP1	2.0	6.6	2.8	1.6
33	AIP2	2.1	5.2	2.0	2.1
34	Fasl	–2.5	10.7	7.2	15.6
35	Fadd	5.8	–2.6	1.8	–10.2
36	Tnfsf10 (TRAIL)	–1.3	9.3	4.3	8.2

# 1–4: Accessory and costimulatory molecules

# 5–11: Toll-like receptors (TLRs) and TLR signaling

# 12–17: Nod1/Nod2 and signaling genes

# 18–26: Inflammasomes

# 27–36: Apoptosis Receptors

### Lethal ehrlichiosis is associated with pro- and anti-inflammatory cytokines, chemokine storm, and a reduced Th1 response

**Figure 1:**
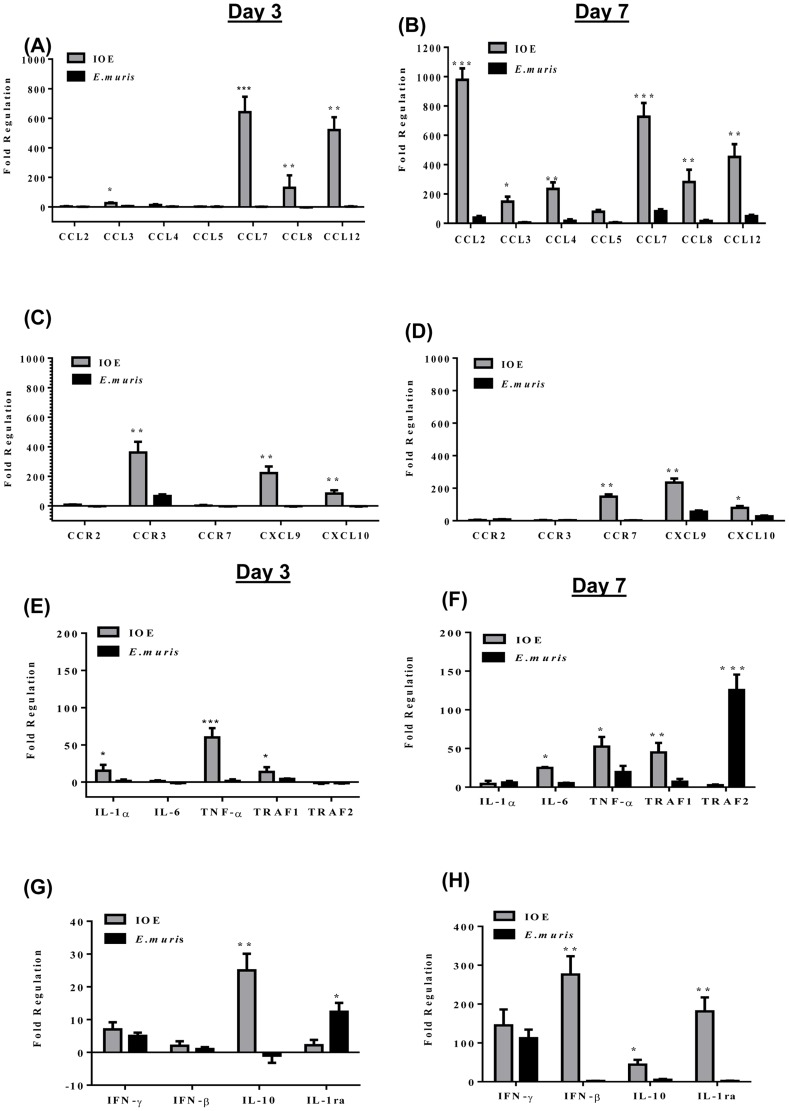
Lethal *Ehrlichia* infection induces higher expression of T-cell and NK cell chemokines and pro- and anti-inflammatory cytokines. The expression levels of several genes in the livers of lethally (IOE) and nonlethally (*E. muris*) infected mice were examined on days 3 (A, C, E, G) and 7 (B, D, F, H) p.i. by real time PCR. A-D show higher expression of chemokines in lethally than nonlethally infected mice. E-H show changes in pro-inflammatory cytokine gene expression. Data presented as fold regulation, showing gene expression differences in lethally (IOE) and nonlethally (*E. muris*) infected mice, normalized to housekeeping genes and relative to gene expression in naive mice. Data shown represent the mean ± SD of individual liver samples with three mice/group. Data represent two independent experiments (* P≤ 0.05, * P≤0.01, *** P≤0.001).

We have recently shown that fatal ehrlichiosis in humans is associated with higher serum levels of chemokines and pro-inflammatory cytokines than detected in patients with mild ehrlichiosis [17], [23]. Thus, we examined whether similar events exist in murine models of fatal and mild ehrlichiosis. We found that lethally infected mice express significantly elevated levels of several chemokines/chemokine receptors compared to naïve and nonlethally infected mice on day 3 p.i., which include *CCL3/MIP-1α*, *CCL4/MIP-1β*, *CCL6*, *CCL7/MCP-3*, *CCL8/MCP-2*, *CCL9*, *CCL12*, *CCL24*, *CCR2*, *CCR3*, *CXCL5*, *CXCL9/Mig*, *CXCL10/IP-10*, and *CXCL13* (Table 1 and Fig. 1A and 1C). In addition to their roles as chemoattractants for macrophages, T cells, NK cells, and granulocytes, most of these chemokines also contribute to the activation of macrophages and T cells. In addition, CXCL10 and CXCL9 chemokines are induced by IFN-γ. On day 7 p.i., in addition to chemokines upregulated on day 3 p.i., lethal infection further induced higher expression of *CCL2*, *CCL6*, *CCL11*, *CCL19*, *CCR7*, *CXCR1*, and *CXCL11* compared to nonlethal infection (Table 1 and Fig. 1B and 1D).

Compared to naive or nonlethally infected mice, lethally infected mice have increased expression of *tnf-α* at early and late stages of infection, which was associated with upregulation of *traf1*, but not *traf2* (Fig. 1E and 1F). TRAF1 and TRAF2 proteins are members of TNF receptors-associated protein family and they mediate signal transduction from various receptors of TNFR superfamily. Expression of the pro-inflammatory *Il-1a* was only elevated in lethally infected mice at early, but not at late, stages of infection (Fig. 1E and 1F). *IL-6* expression was not significantly upregulated in either infection group on day 3 p.i. but was highly significantly upregulated on day 7 p.i. in response to lethal infection than nonlethal infection (Table 1 and Fig. 1E and 1F). Higher expression of *tnf-α* in lethally infected mice was also associated with upregulation of anti-inflammatory *il-10* on days 3 and 7 p.i. compared to nonlethally infected and naive mice (Table 1 and Fig 1G and 1H). Notably, differential overexpression of *il-10* during lethal infection did not influence global *ifn-γ* expression, which was comparable in both lethally and nonlethally infected mice on days 3 and 7 p.i. (Table 1 and Fig. 1G and 1H). However, the *ifn-γ/il-10* ratio was lower in lethally compared to nonlethally infected mice on day 3 p.i. (1/5 vs. 5) and on day 7 p.i. (3.5 vs. 22). Since IL-10 and IFN-γ have suppressor and stimulatory effects on macrophages activation and Th1 responses, respectively, the higher ratio of *ifn-γ/il-10* is a better indication of protective immunity against intracellular pathogens such as *Ehrlichia* than the level of each cytokine alone. Consistent with our previous studies [17]–[21], the expression of Th2 or suppressive cytokines, such as *il-4*, *il-13*, or *TGF-β* was negligible in all groups of mice (data not shown).

Our data also showed that lethal IOE infection induced significantly higher upregulation of IFN-β in the liver than that induced by nonlethal *E. muris* infection (Fig. 1G and 1H). Although IFN-βis known for its anti-viral effect, it promotes induction of chemokines secretion, maturation of dendritic cells and activation of cytotoxic NK cells and is associated with inflammasome activation and regulation of IL-1β secretion [24], [25], [33]. Further, the expression of IL-1receptor antagonist (*il-1ra*) was significantly upregulated in nonlethal infection on day 3 p.i., while it was upregulated in lethally infected mice on day 7 p.i. only (Table 1 and Fig. 1G and 1H). IL-1ra is a natural antagonist of IL-1α and IL-1β signaling that prevents uncontrolled immune activation by IL-1α/β through competitive binding to the IL-1 receptor.

### Lethal ehrlichiosis is associated with activation of inflammasomes

**Figure 2:**
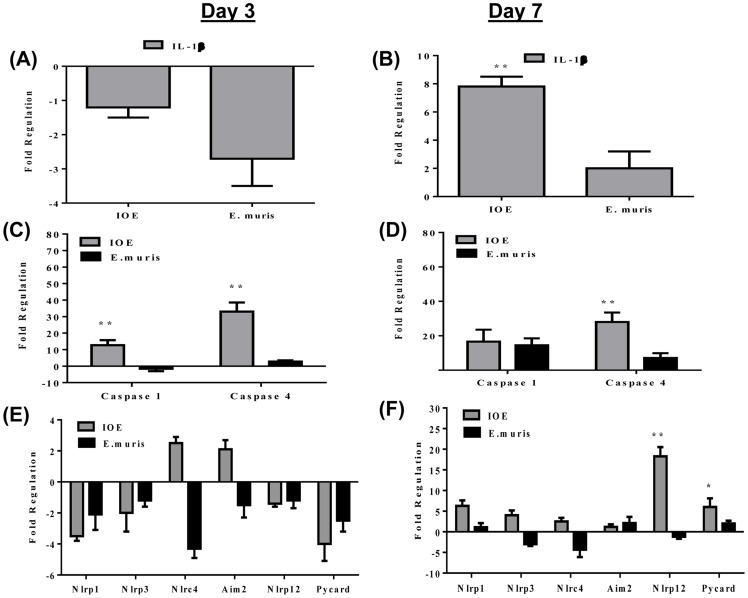
Lethal *Ehrlichia* infection differentially activates the inflammasome compared to nonlethal infection. The expression levels of inflammasome-linked pro-inflammatory cytokine IL-1β are reduced on day 3 p.i. (**A**) but augmented on day 7 p.i. (**B**) in lethally/IOE infected mice. (**C)** and (**D)** show differential induction of caspase 1 and 4 expression on days 3 and 7 p.i. with IOE (lethal) and *E. muris* (nonlethal) infection. (**E)** and (**F)** show differential expression of inflammasome components during lethal and nonlethal infections on day 3 and 7 p.i., respectively. Data shown represent the mean ± SD of individual liver samples with three mice/group. Data represent two independent experiments (* P≤ 0.05, * P≤0.01).

We have previously shown that lethal ehrlichiosis in mice is associated with increased *il-18* production relative to nonlethal infection, suggesting inflammasome activation. Lack of IL-18/IL-18R interaction protected mice from lethal *Ehrlichia* infection, which revealed a detrimental role of IL-18 in this disease process [46]. Our data here show that lethal infection also induced higher expression of *il-1β* on day 7 (Fig. 2B), but not day 3 (Fig. 2A), when compared to nonlethally infected mice. Late *il-1β* expression in lethally infected mice correlated with an early higher expression of *caspase 1* and *caspase 4* (also known as *caspase 11*) on day 3 p.i. compared to nonlethal infection (Fig. 2C). The expression of *caspase 1* was similar in both groups of mice on day 7 p.i., whereas the expression of *caspase 4* remained higher in lethally infected mice than in nonlethally infected mice (Fig. 2D).

Although lethal infection induced higher levels of *caspases 1 and 4* on day 3 p.i., we detected downregulation of several inflammasomes genes (*nlrp1*, *nlrp3*, *nlrc12)* or slight changes in the expression of several inflammasomes (*nlrc4* and *aim2*) in both mice groups at that time (Table 2 and Fig. 2E). However, on day 7 p.i., the liver transcriptional profile showed higher levels of *nlrp1* and *nlrp12* in lethally infected mice, whereas *nlrp3* and *nlrc4* transcript levels were equally increased in both groups of infected mice compared to naïve mice (Table 2 and Fig. 2F). Transcripts for the adaptor molecule, Pycard (PYD and CARD containing domain), were more upregulated in lethal infection on day 7 p.i. than (Fig. 2F). Together, these data suggest that lethal—but not nonlethal—infection is associated with inflammasome activation.

### Upregulation of TLR2 and Nod2 signaling during late stage of lethal *Ehrlichia* infection

**Figure 3:**
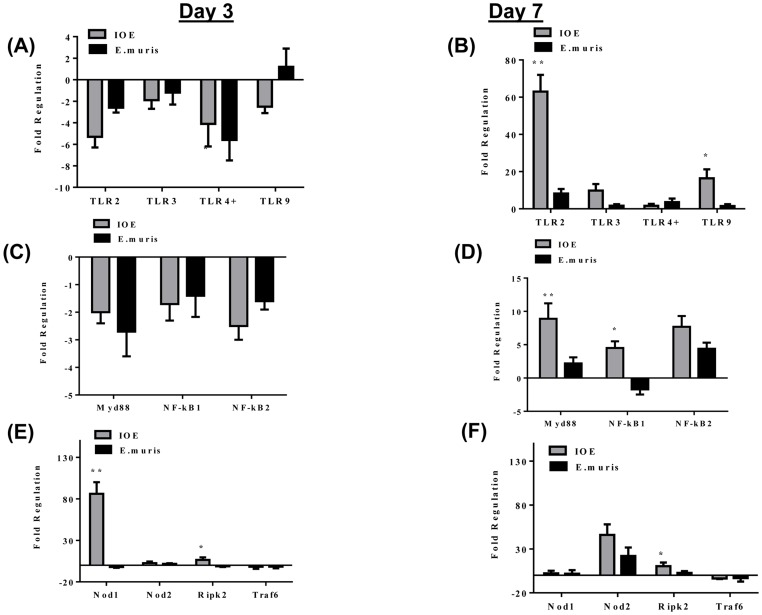
Differential expression of TLR and NOD genes and downstream signaling molecules during lethal and nonlethal *Ehrlichia* infection. The expression of TLRs (A and B), transcription factors (C and D), and Nod1 and 2 proteins and their downstream signaling molecules (Ripk2 and TRAF6) ( E and F) were examined on days 3 and 7 following lethal (IOE) and nonlethal (*E. muris*) infection. The expression of TLR2 on day 7 p.i. with IOE was much pronounced than that of other TLRs. The expression of downstream signaling molecules MyD88 and NF-κB was significantly upregulated on day 7 p.i. during IOE infection compared to *E*. *muris* infection. Nod1 was differentially upregulated on day 3, and Nod2 was differentially upregulated on day 7 after IOE infection. Data shown represent the mean ± SD of individual liver samples with three mice/group. Data represent two independent experiments (* P≤ 0.05, * P≤0.01).

Host cells express a variety of PRRs that recognize different microbial molecular patterns, among which are extracellular and endosomal TLRs and cytoplasmic Nod1 and Nod2 [24]-[28]. Our data show that lethal and nonlethal *Ehrlichia* infection downregulated most Toll-like receptors (*tlr2*, *tlr3*, *tlr4*, and *tlr9*) on day 3 p.i. (Table 2 and Fig. 3A). However, lethally infected mice had a significant upregulation of these TLRs on day 7 p.i., mainly *tlr2* (Fig. 3B), which correlated with significant upregulation of *myd88* and *nf-κb1* when compared to nonlethally infected and naïve mice (Table 2 and Fig. 3C and 3D). Levels of transcripts of other adaptor proteins (e.g., *trif*, *tram*, and *tirap*) in both groups of infected mice were not different from those in naïve mice (data not shown), suggesting that MyD88 is the main protein involved in TLR signaling during ehrlichial infection.

Analysis of intracellular PRRs showed that *nod1* was upregulated 80-fold in lethally infected mice (Fig. 3E), with no significant changes in expression in nonlethally infected mice on day 3 p.i. No change in *nod2* expression was detected in either group of mice on day 3 p.i. (Fig. 3E). However, lethal infection induced higher expression of *nod2* on day 7 p.i. compared to nonlethal infection (Table 2 and Fig. 3F). Activated Nod-2 recruits Ripk2, which activates NF-κB by promoting the ubiquitination of the inhibitor of nuclear factor kappa-B kinase (IKK) subunit of the Ikappa-B kinase complex. Dominant-negative TRAF6 is known to inhibit Ripk2-mediated activation of NF-κB. Our data show that *ripk2* expression was not significantly increased (only 1.8-fold) during nonlethal infection, but was increased approximately 10-fold during lethal infection on day 7 p.i. (Fig. 3F). These data suggest that Nod2 ligation can lead to NF-κB activation in lethally—but not in nonlethally—infected mice. Levels of *traf6* did not significantly differ in either infected group compared to naïve mice (Fig. 3E and 3F).

### Contribution of Tlr2 and Nod2 to *Ehrlichia*-induced immunopathology and bacterial clearance

**Figure 4:**
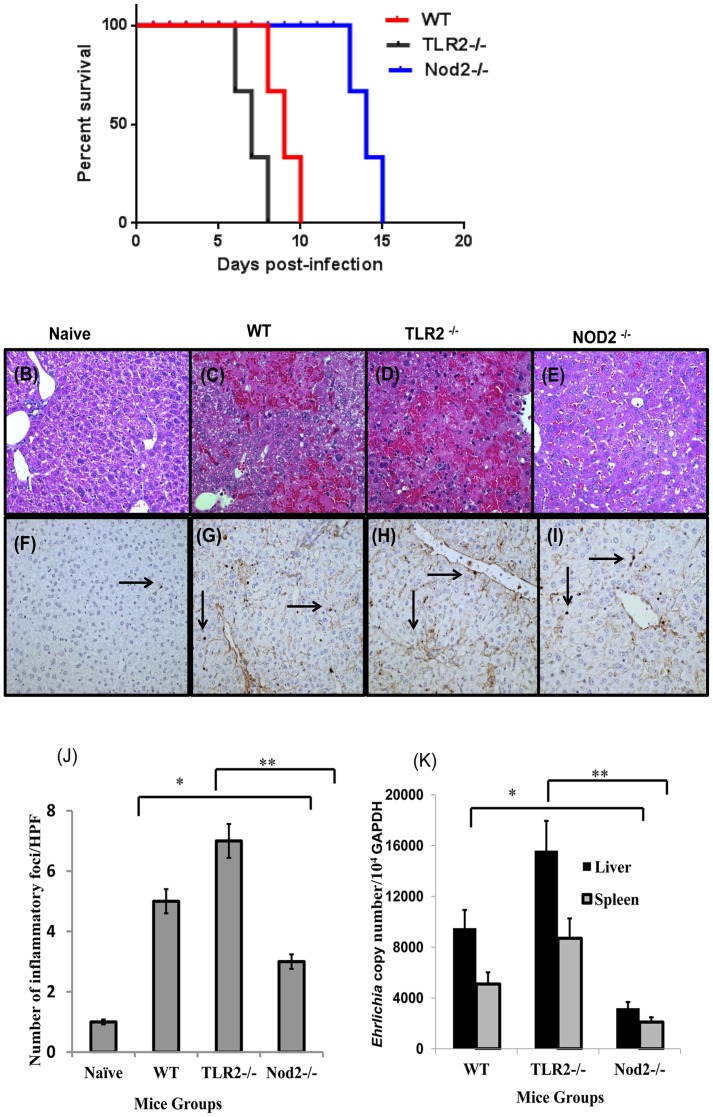
Enhanced resistance of Nod2^-/-^ mice to lethal ehrlichiosis compared to infected wild type and TLR2^-/-^ mice. (A) Survival of WT, TLR2^-/-^ and Nod2^-/-^ mice over 15 days after i.p. infection with high dose of IOE. The data shown represent one of two independent experiments with a total of 6 mice/group. Liver sections from naïve (B and F), IOE-infected WT mice (C and G), IOE-infected TLR2^-/-^ mice (D and H), and IOE-infected Nod2^-/-^ mice (E and I) harvested on day 7 p.i. are stained with H&E. Original magnification for H&E images was 20× and for TUNEL assays was 40×. H&E staining shows that IOE-infected Nod2^-/-^ mice had significant decreases in necrosis compared to infected WT and TLR2^-/-^ mice (arrowheads). TUNEL assay reveals slightly decreased numbers of apoptotic cells (arrows) in Nod2^-/-^ mice with approximately 4–7 apoptotic cells observed per HPF compared with to 6–10 apoptotic cells per HPF for the infected WT and TLR2^-/-^ mice. Uninfected control mice had only one apoptotic cell/HPF. The data shown are from a representative mouse from each group (n = 4) and are from one of three independent experiments with similar results. J) Data show the quantitative analysis of the number of inflammatory foci/HPF determined by H&E staining in different groups of mice. K) shows bacterial burden on day 7 p.i. in the livers and spleens of different groups of mice determined by quantitative real time PCR. The copy number of IOE was normalized to the housekeeping gene GADPH. Bacterial burdens in livers and spleens were lower in IOE-infected Nod2^-/-^ mice compared to WT mice was but were significantly higher in IOE-infected TLR2^-/-^ mice compared to WT mice. Data are expressed as means ± SD with three mice/group and are representative of three independent experiments.

Having observed that lethal IOE infection differentially modulates *tlr2* and *nod2* levels, we decided to elucidate the contributions of TLR2 and Nod2 to the pathogenesis of fatal ehrlichiosis. We infected TLR2^-/-^ and Nod2^-/-^ mice with lethal doses of IOE and compared the outcomes of infection to similarly infected wild type (WT) mice and naïve mice of both strains. Consistent with previous reports [17], WT mice were highly susceptible to lethal IOE challenge where six of six WT mice succumbed to infection on days 9 and 10 p.i. Notably, while TLR2-/- had increased susceptibility to IOE infection with six of six mice succumbed on days 7 and 8 p.i., all Nod2^-/-^ mice survived till days 15 p.i. (Fig. 4A).

Consistent with our previous reports, compared to naïve mice (Fig. 4B and 4F) IOE-infected WT mice developed focal hepatic necrosis and apoptosis on day 7 p.i. (Fig. 4C and 4G). In contrast, IOE-infected Nod2*^-/-^* mice had no evidence of necrosis (Fig. 4E) and presented with fewer inflammatory foci in the liver (Fig. 4J). On the other hand, IOE-infected TLR2^-/-^ mice developed extensive necrosis (Fig. 4D) and inflammatory foci (Fig. 4J) compared to infected Nod2^-/-^ and WT mice on day 7 p.i. There was a slight decrease in number of apoptotic cells in Nod2^-/-^ mice compared to WT and TLR2^-/-^ mice on day 7 p.i. (Compare Fig. 4I to Figs. 4G and 4H). Interestingly, lack of Nod2 enhanced bacterial clearance in different organs, and absence of TLR2 increased bacterial burden when compared to infected WT mice on day 7 p.i. (Fig. 4K).These results collectively suggest that TLR2 and Nod2 play distinct protective and detrimental roles during ehrlichiosis, respectively.

### Lack of Tlr2 or Nod2 influence innate immune responses against *Ehrlichia*

**Figure 5:**
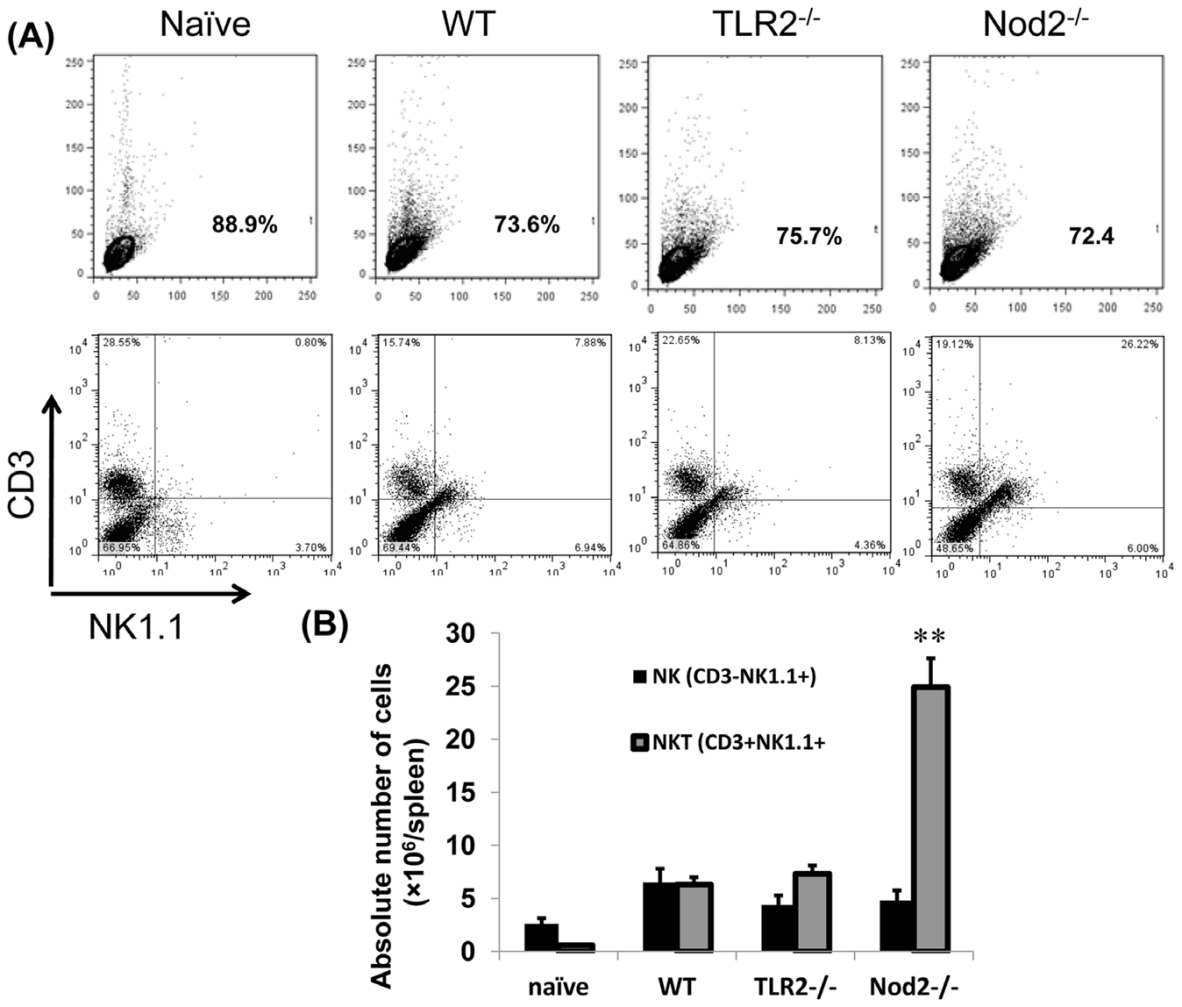
Enhanced resistance to *Ehrlichia* infection in Nod2^-/-^ mice is associated with increased expansion of splenic NKT—but not NK—cells. WT C57BL/6, TLR2^-/-^, and Nod2^-/-^ mice were infected with a high dose of IOE. Splenocytes were harvested from all mice groups on day 7 p.i. and were analyzed directly by flow cytometry. (A) and (B) show increased percentages and absolute numbers, respectively, of protective NKT—but not pathogenic NK—cells on days 7 p.i in Nod2^-/-^ mice compared to WT and TLR2^-/-^ mice. (A) Dot plot data are from one representative mouse from each group (n = 4), and the numbers indicate the percentage of gated cells within each quadrant. (B) The mean ± SD of absolute numbers of cells/spleen is presented with three mice/group. The data are representative of three independent experiments.

**Figure 6:**
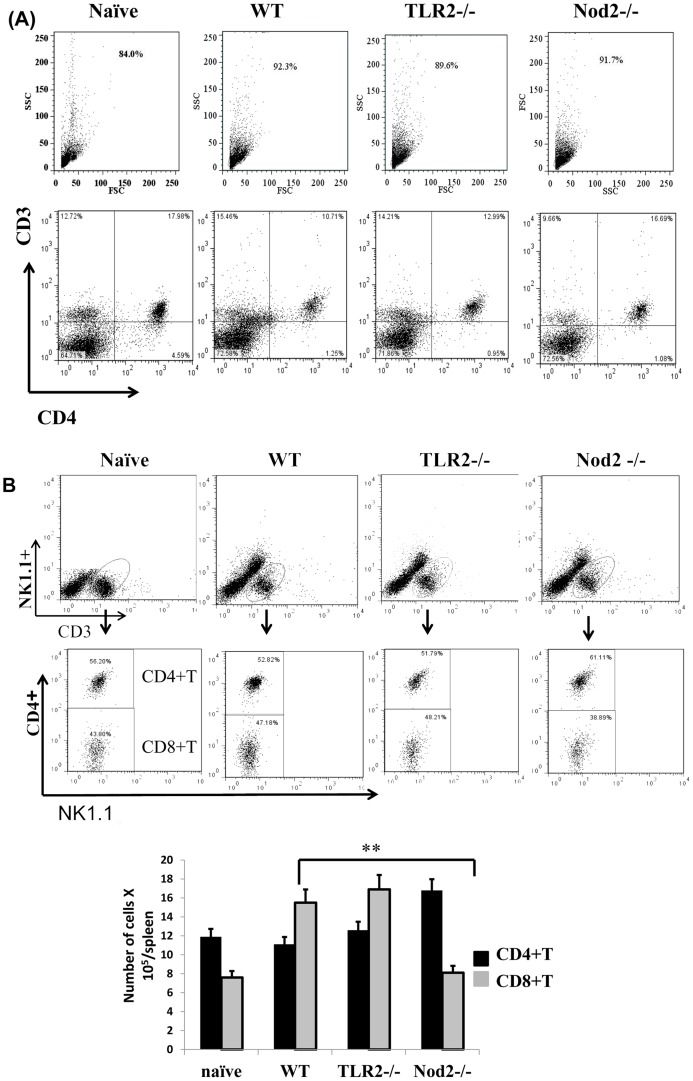
Lack of Nod2 increased number of protective CD4^+^ T cells and decreased frequency of CD8^+^ T cells during severe *Ehrlichia* infection. Spleen cells were harvested from IOE-infected Nod2^-/-^, TLR2^-/-^, and WT mice on day 7 p.i., and cells were analyzed directly *ex vivo* to determine the frequency of CD4^+^ and CD8^+^ T cells. (A) The dot plot shows the percentage of CD3^+^CD4^+^ T cells in naïve mice and IOE-infected WT, TLR2^-/-^, and Nod2^-/-^ mice. (B) CD3^+^ cells were gated and further analyzed for expression of CD4 and NK1.1. NK1.1^-^ cells are thus divided into CD4^+^ T cells (upper quadrant) and CD8^+^ T cells (lower quadrant). Nod2^-/-^ mice have higher percentages of CD4^+^ T cells but lower percentages of CD8^+^ T cells compared to other groups of mice. (C) The absolute numbers of CD4^+^ and CD8^+^ T cells in the four groups of mice. Dot plot data are from a representative mouse from each group. The absolute number of cells represents the means ± SD with three mice/group and is representative of three independent experiments.

**Figure 7:**
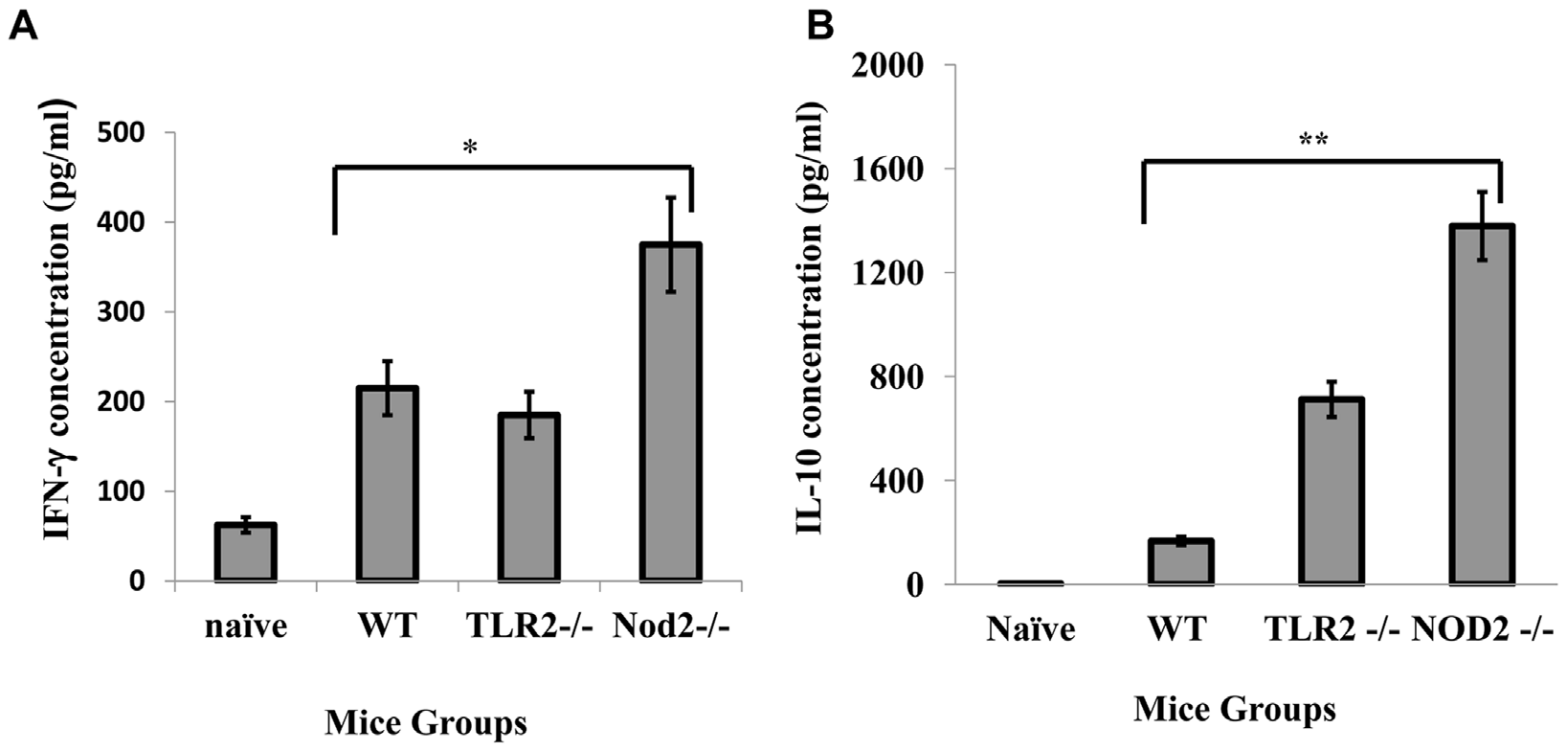
Enhanced resistance to *Ehrlichia* infection in Nod2*^-/-^* mice is associated with increased IFN-γ and IL-10 production. WT C57BL/6, TLR2^-/-^, and Nod2^-/-^ mice were infected with IOE. Splenocytes were harvested on day 7 p.i. and stimulated *in vitro* with IOE antigens. At 48 hours after *in vitro* antigen stimulation, the supernatant was collected and examined for IFN-γ (**A**) and IL-10 (**B**) by enzyme-linked immunosorbent assay. The data are expressed as the mean ± SD for three mice in each group. The data shown are from one experiment that is representative of three independent experiments.

We next examined the contribution of Nod2 and TLR2 to protective or pathogenic immune responses mediated by different innate and adaptive immune cells. Absence of Nod2 increased the percentage (Fig. 5A) and absolute number (Fig. 5B) of NKT as well as percentage (Fig. 6 A and 6B) and absolute number of CD4^+^ T cells (Fig. 6C) in the spleens of infected mice compared to infected WT and TLR2^-/-^ mice. NKT cells and CD4+T cells mediate elimination of intracellular ehrlichiae as shown before (11, 17-23). Although lack of Nod2 did not influence the frequency of pathogenic NK cells, it significantly decreased the percentage and absolute number of pathogenic CD8^+^ T cells when compared to infected WT and TLR2^-/-^ mice (Fig. 6B and 6C). No significant difference was observed in the total number of NK, CD4^+^ T cells, and CD8^+^ T cells between TLR2^-/-^ and WT mice. Interestingly, enhanced resistance and attenuated pathology in IOE-infected Nod2^-/-^ mice correlated with increased antigen-specific production of IFN-γ and IL-10 in the spleen on day 7 p.i. compared to IOE-infected WT and TLR2^-/-^ mice (Fig. 7A and 7B), suggestive of an enhanced Th1 and anti-inflammatory immune responses.

## Discussion

In this study, we provide a detailed genome-wide microarray analysis of whole liver during mild and fatal ehrlichiosis, which offers a revealing new perspective on host responses during the course of nonlethal and lethal *Ehrlichia* infection. *Ehrlichia chaffeensis*, the causative agent of HME, leads to disease in Severe Combined Immunodeficiency (SCID) mice but not in immunocompetent mice [17], [47]. However, several genes identified in our study are consistent with prior reports that profiled the liver of SCID mice infected with different human isolates of *E. chaffeensis*, each belonging to a different genogroup [12], [48]. This suggested that: 1) innate immune responses play a unique role in outcome of *Ehrlichia* infections; and 2) host responses in our murine models of HME using other *Ehrlichia* species (*E. muris* and IOE) mimic the host responses to human *E. chaffeensis* isolates in SCID mice, thus these models are optimal for further analysis of innate and adaptive immune responses during ehrlichiosis in immunocompetent host.

A notable element within the liver transcriptome profile was broad involvement of multiple pro-inflammatory interleukin and TNF family members and their receptors. However, unlike LPS-positive Gram-negative bacteria, severe infection with LPS-negative *Ehrlichia* induced a concomitant upregulation of both pro-inflammatory cytokine genes and key anti-inflammatory genes, such as *il-10* and *il-1ra*. In experimental models of non-infectious inflammatory diseases, IL-10 knockout mice develop severe colitis, whereas systemic administration of rIL-10 can prevent development of colitis [49], [50]. IL-10 mediates an anti-inflammatory function and prevent pathology during infections with intracellular pathogens; however, it also inhibits IFN-γ-mediated activation of phagocytic cells and suppresses the differentiation of protective CD4^+^ Th1 cells, thus inhibit effective elimination of intracellular bacteria [50], [51]. The correlation between late, but not early, expression and production of IL-10 in *E. muris*/ nonlethally infected mice and IOE-infected Nod2^-/-^ mice and survival, protective immunity, minimal pathology and high IFN-γ:IL-10 ratio at early and late stages of infection, suggests that IL-10 may control excessive inflammatory responses and thus inhibits immunopathology without negatively affecting IFN-γ mediated bacterial elimination. On the other hand, production of IL-10 and lowered IFN-γ:IL-10 ratio during early and late stages of lethal IOE infection (Fig. 1E and F) could account for the inhibition of effective bacterial elimination and higher bacterial burden, which in turn would stimulate excessive inflammation that cannot be controlled by IL-10. Similar to the anti-inflammatory function of IL-10, IL-1ra binds to IL-1 receptor I but fails to trigger signal transduction, thereby acting as a competitive inhibitor of IL-1α and IL-1β [52], [53]. Our data show that IL-1ra was upregulated early during nonlethal infection, which correlated with less inflammation and minimal pathology. On the other hand, IL-1ra was upregulated at a late stage of lethal infection but was associated with severe inflammation and pathology. Thus, it appears that late, but not early, induction of IL-1ra during lethal infection may represent an unsuccessful attempt by the host to decrease the excessive inflammation. In contrast, early induction of IL-10 appears to play detrimental roles in host response against *Ehrlichia*. More importantly, the ratio between pro- and anti-inflammatory cytokines and differential kinetics of their production during course of lethal and nonlethal ehrlichial infection are important factors that seems to govern the outcome of infection, degree of pathology, and host immune responses during mild and severe ehrlichiosis.

Immunopathology in lethally infected mice is associated with significant infiltration of NK and CD8^+^ T cells at sites of infection (liver, lung, and peritoneum), which is known to cause tissue injury [17]–[23]. Our data suggest that the development of immunopathology could be attributed to the upregulation of chemokines and their relevant receptors and the recruitment of CD8^+^ T cells, monocytes, and NK cells in the liver of lethally, but not nonlethally, infected mice. Monocytes and NK cells respond to CCL2 (monocyte chemotactic protein [MCP]). CXCL9, CXCL10, and CXCL11 (ligands for the CXCR3) mediate migration of resting human NK cells. T cells and neutrophils respond to CCL5 (RANTES) and CXCL8 (IL-8), respectively [54], [55]. Overproduction of CCL2 in the liver of murine cytomegalovirus was found to be dependent on IFNα/β [56], [57]. We show here that lethal IOE infection dramatically increased expression of *ifn-β* compared to nonlethal *E. muris* infection. Thus, it is possible that type-1 IFN could be the inducing factor responsible for production of this extensive array of chemokines during fatal *Ehrlichia* infection, which in turn enhances migration of inflammatory and immune cells to the sites of infection thus causing immunopathology.

Our data show for the first time that lethal infection is associated with the upregulation of surface and intracellular PRRs, including TLR2, Nod1, and Nod2. TLR2 binds to several microbial ligands, including bacterial lipopeptides and endogenous damage-associated molecules like heat shock proteins or high-mobility group box-1 [58]-[60]. Nod1 binds bacterial peptidoglycan (PGN) and other secreted bacterial outer membrane proteins that access the cytosol. Previous studies have shown that *Ehrlichia* lacks major genes for PGN synthesis. However, a recent study demonstrated that *Ehrlichia* encodes a low-molecular-weight penicillin-binding protein homolog, which is one of the genes of PGN synthesis conserved in *Ehrlichia* and other members of the family Anaplasmataceae [61]. Thus, it is possible that PGN-derived molecules are processed in the phagosome where *Ehrlichia* resides and are then injected into the cytoplasm via a type IV secretion system or other unknown secretory pathway(s) where they bind and activate Nod proteins.

Why lethal *Ehrlichia* infection highly increase Nod1 expression early in infection is not yet clear. However, recent studies have demonstrated that Nod1 signals, together with TLR2 signals, within CD8^+^ T cells can lead to increased proliferation and effector function of CD8^+^ T cells that are activated via TCR ligation. Thus, Nod1 and TLR2 both function as costimulatory receptors [62]. Other studies have shown that Nod1, acting in conjunction with Nod2, enhances the cross-priming and activation of antigen-specific cytotoxic CD8^+^ T cells by CD8α dendritic cells [60]. Thus, it is possible that an early upregulation of Nod1 followed by upregulation of Nod2 could contribute to the induction of pathogenic cytotoxic CD8^+^ T cells during *Ehrlichia*-induced shock. Indeed, our data demonstrate that the absence of Nod2 attenuated immunopathology (Fig. 4A and B) and enhanced protective immunity and bacterial clearance (Fig. 4E). Enhanced resistance of Nod2^-/-^ mice to lethal infection correlated with a reduction in the frequency of CD8^+^ T cells (Fig. 6B and 6C). However, the effect of Nod1/2 on adaptive immunity against *Ehrlichia* appears to be independent of TLR2, because TLR2^-/-^ mice were more susceptible to *Ehrlichia* infection than wild type mice (Fig. 4). Thus, we postulate that Nod2 may promote cross-priming of CD8^+^ T cells. Recently, we showed that lethal infection enhanced the expression of MHC class II—but not class I—molecules on dendritic cells, suggesting that induction of CD8^+^ T cells occur via cross-presentation during lethal ehrlichiosis [46]. Enhanced resistance in Nod2^-/-^ could be also due to effective protective immunity as evidenced by increased number of NKT and CD4^+^ T cells and elevated IFN-γ production (Fig 5B, 6C and 7A). Together, these data suggest that Nod2 contributes to the pathogenesis of fatal ehrlichiosis, possibly via mediating the induction of pathogenic CD8^+^T cells as well as inhibiting protective NKT and antigen-specific CD4^+^Th1 cell producing IFN-γ.

Nod2 binds to pro-caspase 1 and mediates the activation of the inflammasome [60]. Recent study suggested that *Chlamydia trachomatis,* other obligate intracellular bacteria activate inflammasome, which in tun support the bacterial growth within epithelial cells [63]. Thus, another mechanism that could account for effective bacterial elimination in IOE-infected Nod2^-/-^ mice is due to impaired inflammasome activation. In support of this possibility, we found that the responses of IOE-infected Nod2^-/-^mice mimicked the phenotype of IOE-infected IL-18R^-/-^ mice (46), suggesting a common mechanism that involves inflammasome activation. Indeed, our data here shows a differential upregulation of IL-1β, caspase 1, caspase 4, and inflammasome proteins in lethally infected mice, further support a detrimental role of inflammasome activation either in mediating pathology or impairing intracellular bacterial elimination during severe and fatal ehrlichiosis.

Finally, our data reveal for the first time that TLR2 plays a protective role by enhancing intracellular bacterial elimination. Studies have shown that TLR2 and Nod2 cross-regulate the functions of one another, owing to the fact that they recognize the same bacterial molecule (i.e., surface-bound and secreted components of bacterial PGN, respectively) [59], [60], [62]. These contrasting effects of TLR2 and Nod2 on host defense against *Ehrlichia* are thus perplexing. However, it is possible that PGN-activated TLR2 signals enhance the intracellular microbicidal functions of phagocytic cells or that this effect could be negatively regulated by PGN-mediated activation of Nod2 in wild type mice. Thus, in the absence of Nod2 (such as in Nod^-/-^ mice), negative regulation of TLR2 is removed, and bacteria are effectively eliminated. In contrast, the lack of TLR2 and unrestricted function of Nods in TLR2^-/-^ mice could lead to uncontrolled IOE infection and immunopathology, which is consistent with the phenotype of IOE-infected Nod2-/- mice (Fig. 4). Previous *in vivo* and *in vitro* studies showed that the lack of Nod2 increased Th1 responses, which mediate activation of intracellular bactericidal functions of macrophages. Interestingly, our data demonstrate that IOE-infected Nod2^-/-^ mice had higher levels of IFN-γ than IOE-infected WT and TLR2^-/-^ mice (Fig. 7D), which could be responsible for the effective bacterial elimination in these mice.

In conclusion, our study indicates Nod2 mediates the dysregulated inflammatory responses and immunopathology during lethal ehrlichiosis and TLR2 mediates effective clearance of ehrlichiae in the absence of Nod2 signals. Our data thus define for the first time unique molecular pathogenic mechanisms that may account for the development of *Ehrlichia*-induced shock. Targeting these pathways could represent a novel immunotherapeutic strategy to combat these important infections and the associated pathology.
